# Prolyl Isomerase Pin1 Regulates the Stability of Hepatitis B Virus Core Protein

**DOI:** 10.3389/fcell.2020.00026

**Published:** 2020-01-31

**Authors:** Mayuko Nishi, Kei Miyakawa, Satoko Matsunaga, Hajera Khatun, Yutaro Yamaoka, Koichi Watashi, Masaya Sugiyama, Hirokazu Kimura, Takaji Wakita, Akihide Ryo

**Affiliations:** ^1^Department of Microbiology, Yokohama City University School of Medicine, Yokohama, Japan; ^2^Isehara Research Laboratory, Technology and Development Division, Kanto Chemical Co., Inc., Isehara, Japan; ^3^Department of Virology II, National Institute of Infectious Diseases, Tokyo, Japan; ^4^Genome Medical Sciences Project, National Center for Global Health and Medicine, Chiba, Japan; ^5^Faculty of Health Sciences, School of Medical Technology, Gunma Paz University, Takasaki, Japan

**Keywords:** virus-host interaction, phosphorylation, prolyl isomerization, lysosome, hepatitis B virus

## Abstract

The dynamic interplay between virus and host proteins is critical for establishing efficient viral replication and virus-induced pathogenesis. Phosphorylation-dependent prolyl isomerization by Pin1 provides a unique mechanism of molecular switching to control both protein function and stability. We demonstrate here that Pin1 binds and stabilizes hepatitis B virus core protein (HBc) in a phosphorylation-dependent manner, and promotes the efficient viral propagation. Phos-tag gel electrophoresis with various site-directed mutants of HBc revealed that Thr160 and Ser162 residues within the C terminal arginine-rich domain are phosphorylated concomitantly. GST pull-down assay and co-immunoprecipitation analysis demonstrated that Pin1 associated with phosphorylated HBc at the Thr160-Pro and Ser162-Pro motifs. Chemical or genetic inhibition of Pin1 significantly accelerated the rapid degradation of HBc via a lysosome-dependent pathway. Furthermore, we found that the pyruvate dehydrogenase phosphatase catalytic subunit 2 (PDP2) could dephosphorylate HBc at the Pin1-binding sites, thereby suppressing Pin1-mediated HBc stabilization. Our findings reveal an important regulatory mechanism of HBc stability catalyzed by Pin1 and may facilitate the development of new antiviral therapeutics targeting Pin1 function.

## Introduction

Virus–host interactions play important roles in virus replication and pathogenesis (Brito and Pinney, 2017). Viruses have evolved a number of ways of hijacking host machinery and cellular regulatory mechanisms to produce progeny viruses, as well as counteracting host immune systems (Mitra et al., 2018). Understanding these elaborate interactions may provide insight into the basic host elements indispensable for the viral life cycle, as well as antiviral host factors counteracting viral propagation. Moreover, the accumulation of information relevant to the molecular basis of virus–host interactions could be of great use in the development of new antiviral strategies.

Hepatitis B virus (HBV), a globally leading infectious agent, is the main cause of hepatitis, liver cirrhosis, and hepatocellular carcinoma (HCC) (McMahon, 2005; Baumert et al., 2007). Despite the availability of an HBV vaccine, approximately 350 to 400 million people are constantly infected with the virus in the world (Cryer and Imperial, 2019). Epidemiological studies suggest that persistent HBV infection is the major factor for the development of HCC (Lee and Ahn, 2016; Wang et al., 2017). HCC is a chief cause of cancer-associated deaths, highlighting the requirement for understanding the molecular mechanisms that regulate HBV replication in chronically infected HBV patients.

The genome structure of HBV is composed of circular partially double-stranded DNA, which is approximately 3.2 kb long and encodes four genes designated C (core), X, P (polymerase), and S (surface) (Beck and Nassal, 2007). Among these viral proteins, HBV core protein (HBc) plays pivotal roles in the viral replication processes, acting as the basic unit for capsid assembly, and is involved in HBV genome replication and progeny virion biosynthesis (Zheng et al., 2019). An essential structural element of HBV is the spherical capsid, which consists of multiple copies of a single HBc that contains viral pre-genomic RNA (pgRNA) and polymerase. HBc is a 21.5-kDa protein and composed of two specific domains, the N-terminal self-assembly domain (amino acids 1–140) and the C-terminal arginine-rich domain (CTD, amino acids 150–185) for the nucleic acid–binding (Nassal, 1992; Newman et al., 2003; Steven et al., 2005). The CTD plays an essential role in the specific encapsidation of pgRNA and polymerase during replication. Moreover, the phosphorylation of serine (Ser) or threonine (Thr) residues within the CTD can modulate multiple stages of HBV replication, such as viral core formation and subcellular localization (Diab et al., 2018). Although the accumulated evidence has emphasized the functional significance of the phosphorylation of CTD, it is still unknown whether phosphorylated HBc (pHBc) is subjected to further post-phosphorylation regulation.

The phosphorylation of proteins on serine or threonine residues that immediately precede proline (Ser/Thr-Pro) provides a unique signaling mechanism regulating a plethora of cellular processes, including cell proliferation, differentiation, and cell death (Lu et al., 2002). Peptidyl-prolyl *cis-trans* isomerase NIMA-interacting 1 (Pin1) is a regulator that specifically interact with phosphorylated Ser/Thr-Pro motifs and catalyzes the *cis* and *trans* amide isomer interconversion, leading to the conformational changes of its substrates (Lu and Zhou, 2007). This Pin1-mediated prolyl isomerization can provide further post-phosphorylation modifications that control various protein functions, such as protein stability, catalytic activity, protein–protein interactions, dephosphorylation and/or subcellular localization (Wulf et al., 2005; Lu et al., 2007; Liou et al., 2011; Nakamura et al., 2012). Recent studies have demonstrated that a number of viral proteins are also regulated by Pin1-mediated prolyl isomerization (Kojima and Ryo, 2010).

Here, we demonstrate that Pin1 binds pHBc and regulates its stability to sustain efficient viral replication. Specifically, we show that the targeted inhibition of Pin1 facilitates the prompt degradation of HBc via the lysosomal pathway. Furthermore, using NanoBRET technology, we showed that PDP2 serves as a negative regulator for HBc by selectively dephosphorylating HBc, thereby inhibiting the Pin1–HBc interaction. Our findings reveal an important molecular mechanism of HBc stabilization by Pin1-dependent prolyl isomerization and might provide insight into new antiviral therapeutics targeting Pin1 function.

## Results

### Identification of Phosphorylation Sites in HBc CTD

**FIGURE 1:**
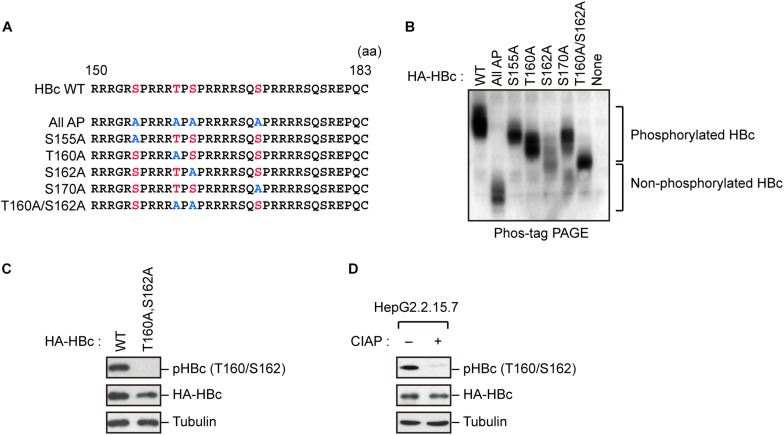
Concomitant phosphorylation of HBc at Thr160 and Ser162. **(A)** Schematic representation of the HBc deletion mutants generated in this study. The sequence of the HBc CTD, with the four major phosphorylation sites (S155, T160, S162, and S170) and alanine substitutions, is shown. **(B)** Mobility shifts of HBc in Phos-tag Gel. HepG2 cells were transfected with plasmids encoding HA-HBc or its site-directed mutants. The transfected cells were harvested at 24 h post-transfection, and cell lysates were subsequently subjected to Phos-tag gel electrophoresis and analyzed by immunoblot analysis with anti-HA antibody. **(C)** Detection of phosphorylation of HBc by phospho-specific antibody. HepG2 cells were transfected with WT HBc or its site-directed (T160A/S162A) mutant for 48 h in the presence of protease inhibitors. Cell lysates were then subjected to immunoblot analysis with anti-phospho HBc (T160/S162), anti-HBc, or anti-α-tubulin antibodies. **(D)** Cell lysates from stably HBV-producing HepG2.2.15.7 cells were treated or not treated with calf intestine alkaline phosphatase (CIAP), and then subjected to immunoblotting with anti-phospho HBc (T160/S162), anti-HBc, and anti-α-tubulin antibodies.

Because HBc CTD contains multiple phospho-acceptor sites at Ser/Thr residues, we generated site-directed mutants in which Ser/Thr residues were replaced by alanine (Figure 1A). The wild-type (WT) HBc and the mutant proteins were expressed in cells, and cell lysates were subjected to Phos-tag polyacrylamide gel electrophoresis followed by immunoblot analysis. In a Phos-tag gel, the migration speed of phosphorylated proteins is reduced, separating them from non-phosphorylated proteins (specifically, the bands shift upward) (Kinoshita et al., 2006). WT HBc exhibited the most prominently shifted broad bands, reflecting its phosphorylation at multiple sites. On the other hand, HBc harboring a T160A or S162A mutation yielded relatively lower molecular weight bands than WT HBc and other site-directed mutants (S155A and S170A). Notably, the T160A/S162A double mutant yielded a much lower molecular weight band, implying that both sites are phosphorylated within HBc (Figure 1B). To further confirm phosphorylation at Thr160 and Ser162, we produced a phospho-specific HBc antibody (anti-pHBc) that exclusively detects phosphorylated Thr160/Ser162. Cells expressing either HA-tagged WT HBc or the T160A/S162A mutant were processed for the immunoblot analysis with anti-pHBc or anti-HA antibody. We observed phosphorylation of HBc only in WT HBc, but not in the T160A/S162A mutant (Figure 1C). Importantly, the phosphorylation signal was also detected in stably HBV-producing HepG2.2.15.7 cells, but this signal was diminished when the cell lysate was pre-treated with calf intestine alkaline phosphatase (CIAP) (Figure 1D). These results indicate that Thr160 and Ser162 are distinct phosphorylation sites within HBc.

### Pin1 Interacts With Phosphorylated HBc

**FIGURE 2:**
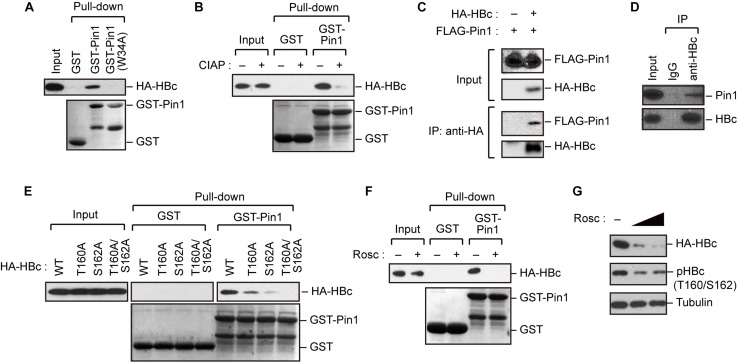
Pin1 interacts with phosphorylated HBc. **(A)** HepG2 cells were transfected with plasmid encoding HBc. After 48 h, cell lysates were subjected to GST pull-down analysis with GST, GST-Pin1, or GST-Pin1W34A mutant followed by immunoblotting with anti-HA antibody. **(B)** Cell lysates derived from HepG2 cells transfected with HBc were treated or not treated with CIAP, followed by GST pull-down analysis as described in **(A)**. **(C)** HepG2 cells were transfected with HA-HBc and FLAG-Pin1 expression vectors. After 48 h, cell lysates were subjected to immunoprecipitation (IP) analysis with anti-HA or non-immunized IgG, followed by immunoblotting analysis with anti-FLAG or anti-HA antibodies. **(D)** HepG2.2.15.7 cell lysates were subjected to IP analysis with anti-HBc or non-immunized IgG, followed by immunoblotting analysis with anti-Pin1 or anti-HBc antibodies. **(E)** Pin1 interacts with HBc via its Thr160-Pro and Ser162-Pro motifs. HepG2 cells were transfected with WT HBc or the indicated mutants for 48 h in the presence of lysosome inhibitors. Cell lysates were then subjected to GST pull-down followed by immunoblot analysis. **(F)** HepG2 cells were transfected with HA-HBc expression plasmid. At 24 h following transfection, cells were treated with roscovitine (Rosc, 50 μM). After 15 h, cell lysates were harvested and subjected to GST pull-down analysis as shown in **(A)**. **(G)** HepG2 cells expressing HA-HBc were treated with roscovitine (Rosc, 25 or 50 μM). After 15 h, cell lysates were subjected to immunoblotting analysis with indicated antibodies.

The results described above indicate that HBc is phosphorylated at Thr160 and Ser162, both of which are potential Pin1-binding sites (pSer/Thr-Pro). We next asked whether Pin1 directly binds to these sites within HBc. To this end, we generated recombinant GST-Pin1 and the WW domain mutant (W34A), which lacks pSer/Thr-Pro binding activity (Zhou et al., 2000). GST pull-down assay with whole-cell lysate from HepG2 cells expressing HA-HBc revealed that HBc co-precipitated with GST-Pin1 but not with GST-Pin1W34A or control GST (Figure 2A). The association between Pin1 and HBc was abolished by pretreatment of the cell lysates with CIAP prior to the GST pull-down analysis (Figure 2B), indicating that Pin1 can only interact with pHBc. The intracellular interaction between HBc and Pin1 was also confirmed by immunoprecipitation analysis where Pin1 was co-precipitated with HA-HBc (Figure 2C). We also observed that endogenous Pin1 could bind HBc in HBV-producing cells (Figure 2D). We next attempted to determine the Pin1-binding sites for HBc. HepG2 cells were transfected with plasmid encompassing HA-HBc or its mutants (T160A, S162A, or T160A/S162A), and then subjected to GST pull-down assay. We found that a single site-directed mutation (T160A and S162A) resulted in the prominent reduction of co-precipitated HBc with GST-Pin1 (Figure 2E). Notably, HBc harboring a double mutation (T160A/S162A) completely lost the ability to bind GST-Pin1 (Figure 2E). These results illustrate that Pin1 directly interacts with pHBc at Thr160 and Ser162. Although above Phos-tag analysis indicated that S162 is the major site of the Pin1-binding phosphorylation site (Figure 1B), we found that a single site-directed mutant (S162A) still interacted with Pin1 with relatively lower binding activity. Since mutation in both sites (T160A/S162A) completely abolished its Pin1-binding, these results indicate that Thr160 is an another Pin1 binding-phosphorylated residue.

Given that HBc CTD can be phosphorylated by cyclin-dependent kinases (CDKs) (Ludgate et al., 2012), we next asked whether the inhibition of CDKs could affect the Pin1 interaction with HBc. We found that treatment with the broad-spectrum CDK inhibitor roscovitine significantly reduced Pin1–HBc binding along with decreased levels of pHBc (Figures 2F,G), indicating that CDKs contribute to the Pin1–HBc interaction, presumably by mediating the phosphorylation of Thr160/Ser162 residues.

### Pin1 Regulates HBc Stability

**FIGURE 3:**
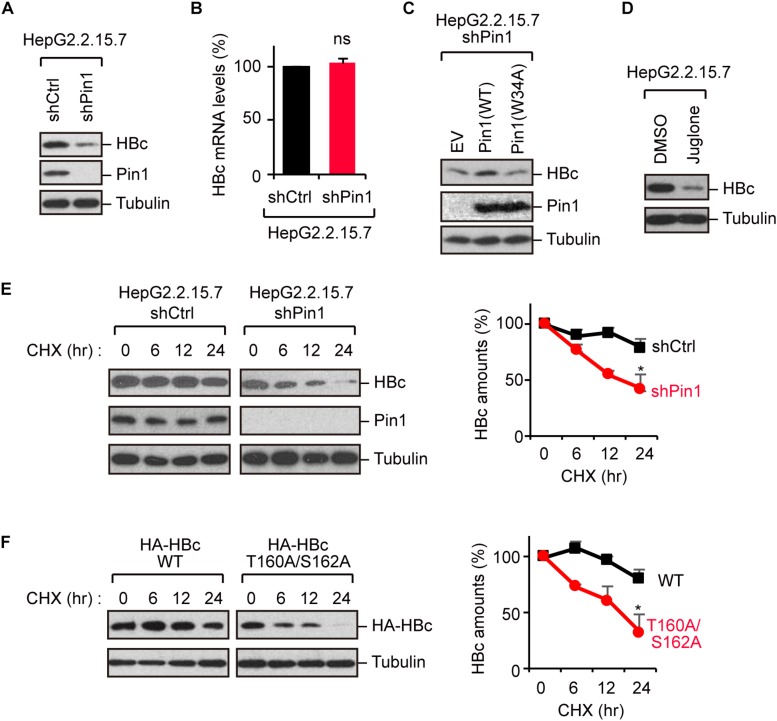
Pin1 regulates HBc stability. **(A)** Lysates from HepG2.2.15.7 cells that were infected with retroviral vectors carrying control shRNA (shCtrl) or Pin1-specific shRNA (shPin1) were immunoblotted with anti-HBc, anti-Pin1, or anti-α-tubulin antibodies. **(B)** Total mRNA from indicated HepG2.2.15.7 cells were subjected to quantitative PCR for HBc mRNA. Data were normalized with the amounts of glyceraldehyde 3-phosphate dehydrogenase (GAPDH). ns, not significant. **(C)** Pin1-depleted HepG2.2.15.7 cells were transfected with empty vector (EV), Pin1WT, or Pin1W34A mutant. After 48 h, cell lysates were subjected to immunoblotting with anti-HBc, anti-Pin1, or anti-α-tubulin antibodies. **(D)** HepG2.2.15.7 cells were treated with either DMSO or 5 μM juglone for 24 h. Cell lysates were then subjected to immunoblot analysis with anti-HBc or anti-α-tubulin antibodies. **(E)** HepG2.2.15.7 cells were treated with 100 μM cycloheximide (CHX) and harvested at the indicated time points, followed by immunoblotting analysis with anti-HBc, anti-Pin1, or anti-α-tubulin antibodies. Quantitative data are shown in the right panel. ^∗^*P* < 0.05, two-tailed unpaired *t*-test. **(F)** HepG2 cells were transfected with WT HBc or the T160A/S162A mutant followed by CHX assay as shown in **(D)**. Quantitative data are shown in the right panel. *P < 0.05, two-tailed unpaired *t*-test.

Because Pin1 is a general regulator of protein stability, it is plausible that Pin1 could stabilize HBc. To test this proposition, we knocked down Pin1 in HepG2.2.15.7 cells by stable transduction of Pin1-specific shRNA. Immunoblot analysis demonstrated that HBc expression was significantly decreased upon Pin1 depletion (Figure 3A). Notably, a parallel experiment showed that HBV mRNA levels were not significantly altered following Pin1 depletion (Figure 3B), indicating post-translational regulation of HBc. The reduced level of HBc was also observed in Pin1-knockdown HepG2.2.15.7 cells, and this reduction was rescued by transient expression of Pin1, but not Pin1W34A (Figure 3C). We also found that the Pin1 inhibitor juglone (Chao et al., 2001) prominently reduced the protein expression of HBc (Figure 3D). Cycloheximide analysis further revealed that the protein stability of HBc was prominently decreased in Pin1-knockdown cells as compared with control cells (Figure 3E). Together, these results suggest that Pin1 inhibition decreases HBc stability, thereby decreasing the HBc protein level in cells.

To further delineate the functional implication of the Pin1–HBc interaction, we investigated the protein stability of the T160A/S162A mutant, which is unable to bind Pin1. Cycloheximide analysis demonstrated that the HBc-T160A/S162A mutant was conspicuously destabilized relative to WT HBc (Figure 3F), confirming that Pin1 indeed regulates the HBc stability by interacting with the phosphorylated Thr160-Pro and Ser162-Pro motifs.

### Pin1 Inhibits Lysosomal Degradation of HBc

**FIGURE 4:**
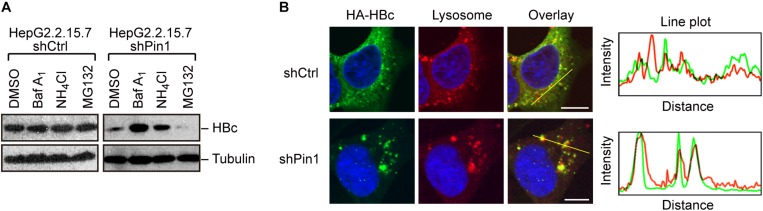
Pin1 inhibits lysosomal degradation of HBc. **(A)** HepG2.2.15.7 cells transduced with shCtrl or shPin1 were treated with the indicated inhibitors for 24 h. Cell lysates were then subjected to immunoblot analysis with anti-HBc or anti-α-tubulin antibodies. The final concentration of inhibitors as follows; BafilomycinA1, 100 nM; NH_4_Cl, 4 mM; MG132, 10 μM. **(B)** HepG2 cells transduced with shCtrl or shPin1 cells were transfected with HA-HBc expression vector. After 24 h, cells were fixed with 3% formaldehyde and immunostained with anti-HA (green), LysoTracker (red), and DAPI (blue). Cells were then subjected to confocal microscopy. Scale bar, 10 μm. Line plots indicate the fluorescence intensity of the left images.

Given that Pin1 stabilizes HBc, we next attempted to clarify the molecular pathway by which HBc degraded. To this end, we utilized chemical inhibitors, bafilomycin and NH_4_Cl (lysosome inhibitors) or MG132 (proteasome inhibitor). Pin1-depleted HepG2.2.15.7 cells were treated with each inhibitor for 24 h, and HBc protein levels were examined by immunoblotting. Our result demonstrated that bafilomycin and NH_4_Cl, but not MG132, prominently reverted the HBc instability upon Pin1 knock-down (Figure 4A), indicating that Pin1 might inhibit the endo-lysosomal degradation of HBc. Immunofluorescence analysis demonstrated that HBc was colocalized with the lysosome, forming cytoplasmic foci in Pin1-knockdown cells whereas control cells exhibited a relatively diffuse pattern of HBc in the cytoplasm without lysosomal co-localization (Figure 4B). Together, these results indicate that Pin1 counteracts the lysosomal degradation of HBc.

### Screening of Phosphatases for Pin1 Bindings Sites Within HBc

**FIGURE 5:**
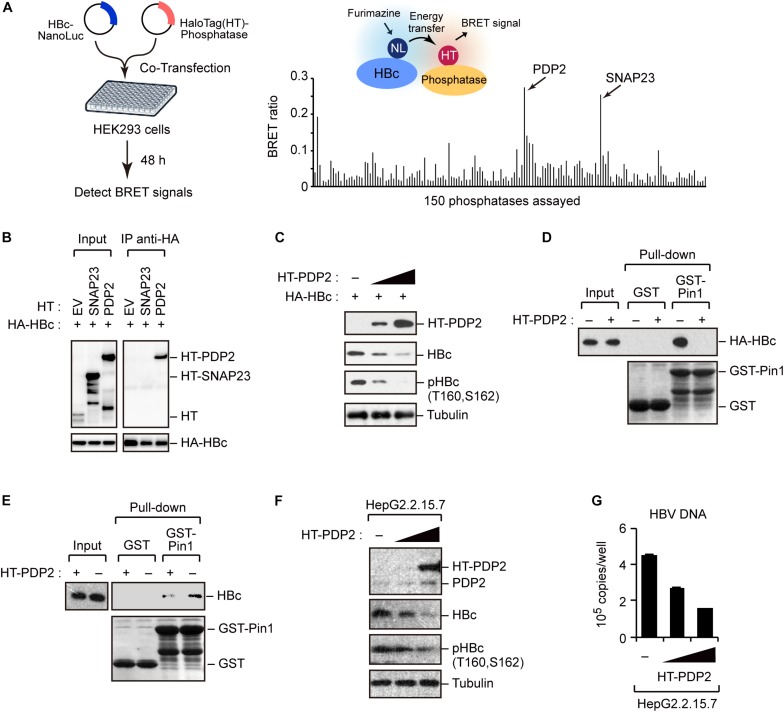
Screening of phosphatases for Pin1 binding sites within HBc. **(A)** NanoBRET-based screen to identify HBc-interacting proteins in living cells. Schematic representation of the NanoBRET-based screening method (left panel). HEK293 cells were co-transfected with NanoLuc-tagged HBc and HaloTag-conjugated phosphatase expression vectors, followed by Halotag-620 ligand and furimazine substrate addition to the cells. If two proteins were within 200 nm of each other, BRET signals were detected. Two candidates with high BRET ratios (>0.2; right panel) were also shown. **(B)** HEK293 cells were co-transfected with HA-HBc together with HT empty vector (EV), HT-SNAP23, or HT-PDP2 and cultured for 24 h in the presence of protease inhibitors. Cell lysates were then subjected to immunoprecipitation with anti-HA antibody, followed by immunoblot analysis with the indicated antibodies. **(C)** PDP2 decreases HBc-T160/S162 phosphorylation. HepG2 cells were co-transfected with the expression vector encoding HA-HBc and Halotag (HT)-PDP2. At 48 h post-transfection, cells were harvested and subjected to immunoblot analysis with the indicated antibodies. **(D,E)** PDP2 interferes with HBc-Pin1 interaction. HepG2 cells expressing HA-HBc and HT-PDP2 **(D)** or HepG2.2.15.7 cells expressing HT-PDP2 **(E)** were lysed and subjected to GST pull-down analysis with GST or GST-Pin1, followed by immunoblot analysis with indicated antibodies. **(F,G)** HepG2.2.15.7 cells were transfected with expression vector encoding HT-PDP2. At 48 h post-transfection, cell lysates were subjected to immunoblot analysis with indicated antibodies. The levels of HBV DNA in the culture supernatants were measured by real-time PCR.

To better understand the regulation of HBc stability, we screened host phosphatases that remove phosphate(s) from pHBc. For this object, we performed the NanoBRET protein–protein interaction assay (Machleidt et al., 2015). This method employs a NanoLuc fusion protein as the bioluminescent donor and a fluorescently labeled HaloTag fusion protein as the acceptor. We cotransfected the NanoLuc-tagged HBc and 150 different HaloTag-conjugated phosphatases into HEK293 cells (Figure 5A, left). At 48 h post-transfection, the BRET signal was visualized, and a BRET ratio >0.2 was used as the threshold. We identified two phosphatases (SNAP23 and PDP2) whose BRET signals were much higher than those of other phosphatases (Figure 5A, right). Accordingly, we focused on SNAP23 and PDP2 for further functional analysis. To investigate the direct interaction of the phosphatases with HBc, we performed immunoprecipitation analysis using HepG2 cells co-transfected with plasmids encoding HA-HBc and either HT-PDP2 or SNAP23. Our result revealed that HBc was co-precipitated with PDP2, but not with SNAP23 (Figure 5B), indicating that PDP2 can physically associate with HBc.

We next asked whether PDP2 could dephosphorylate HBc. HepG2 cells were co-transfected with HA-HBc and HT-PDP2. After 48 h, cells were harvested and cell lysates were subjected to immunoblot analysis Our data demonstrated that PDP2 expression decreased the level of HBc while dephosphorylating it in a dose-dependent manner (Figure 5C). Consistent with this, GST pull-down assay revealed that PDP2 overexpression inhibited the interaction between Pin1 and HBc (Figure 5D). These results were also confirmed in HepG2.2.15.7 cells; PDP2 was able to decrease pHBc and interfere with Pin1-HBc interaction (Figures 5E,F). Of note, we found that PDP2-mediated dephosphorylation of HBc could negatively regulate HBV particle production (Figure 5G). These results together indicate that PDP2-mediated HBc dephosphorylation results in the dissociation of Pin1 from HBc, thereby reducing HBc stability as well as HBV biosynthesis.

### Effect of Pin1–HBc Interaction on HBV Propagation

**FIGURE 6:**
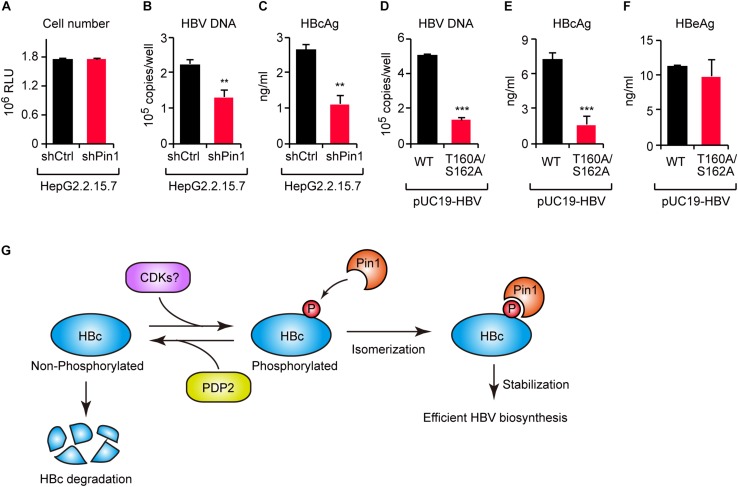
The HBc–Pin1 interaction regulates HBV biosynthesis. **(A)** Cell viability analysis of HepG2.2.15.7 cells stably expressing shCtrl or shPin1. **(B,C)** The levels of HBV DNA **(B)** and HBcAg **(C)** in the culture supernatants of HepG2.2.15.7-shCtrl or HepG2.2.15.7-shPin1 cells were measured by real-time PCR and ELISA, respectively. **P < 0.01, two-tailed unpaired *t*-test. **(D–F)** HepG2 cells were transfected with an HBV molecular clone (pUC19-C_JPNAT) and its site-directed mutant (T160A/S162A). After 24 h, the levels of HBV DNA in the culture the culture supernatants were measured by real-time PCR **(D)**, and the levels of HBcAg **(E)** or HBeAg **(F)** in the culture supernatants were measured by ELISA. ***P < 0.001, two-tailed unpaired *t*-test. **(G)** Schematic representation of the model proposed in this study. CDKs phosphorylate HBc to create Pin1-binding sites. Subsequently, Pin1 stabilizes HBc by preventing its lysosomal degradation, thereby promoting effective HBV biosynthesis. On the other hand, PDP2 dephosphorylates HBc to enhance its degradation.

To investigate the functional role of Pin1 in HBV replication, we attempted to knock down Pin1 in HepG2.2.15.7 cells that can stably secrete viral particles in culture supernatant. We then analyzed HBV DNA and virus core antigen (HBcAg) in the cell supernatant by quantitative PCR and ELISA, respectively. The results illustrated that Pin1 knockdown had no effect on cell proliferation (Figure 6A), but prominently decreased the levels of both viral DNA and HBcAg relative to control cells (Figures 6B,C), indicating a reduction in viral particle production. To further delineate the biological importance of the Pin1–HBc interaction, we tested the efficiency of virus production of HBV encoding WT HBc or its T160A/S162A mutant. HepG2 cells were transfected with an HBV molecular clone (either WT or T160A/S162A), and supernatants were collected to analyze HBV DNA and HBcAg. Amounts of HBV DNA and HBcAg, but not HBeAg devoid of Pin1-binding site, were significantly reduced in the case of the T160A/S162A virus relative to the WT virus (Figures 6D–F). Together, these results indicate that the Pin1 interaction with HBc stabilizes HBc, eventually leading to efficient virus particle production in HBV infected cells.

## Discussion

Viral proteins are required to interact with host proteins to maintain the viral life cycle. Some host proteins act as antiviral factors to restrict viral propagation, whereas others interact with viral proteins in a manner that sustains viral replication. Understanding the molecular operations of the virus-host interaction will aid in identification of new therapeutic targets and to develop antiviral strategies. In this study, we revealed that the peptidyl-prolyl isomerase Pin1 is a potent host factor that binds HBc and facilitates viral biogenesis. Moreover, by screening a phosphatase library, we identified PDP2 as the phosphatase responsible for the dephosphorylation of Thr160/Ser162 residues within HBc. PDP2 counteracts Pin1-mediated HBc stabilization, thereby decreasing virus propagation (Figure 6G). Our current findings shed new light on a virus–host interaction mediated by viral protein phosphorylation and subsequent prolyl isomerization by Pin1.

Protein phosphorylation is a major fashion of post-translational modification and, by modulating intracellular signaling pathways, serves as an essential regulatory event for many cellular processes (Hunter, 1995). Phosphorylated proteins are likely to undergo a novel type of post-phosphorylation regulation by Pin1. Pin1 recognizes phosphorylated serine or threonine residue immediately preceding a proline residue (pSer/Thr-Pro) (Ryo et al., 2003). Following the binding to substrates, Pin1 catalyzes the conformation *via cis-trans* isomerization of the peptide bonds, which alters the catalytic activity, localization, and stability of target proteins (Ryo et al., 2003; Lu and Zhou, 2007). Our current observations show that Pin1 binds to phosphorylated HBc, thereby stabilizing the viral protein. Accordingly, Pin1 inhibition promotes HBc degradation via the lysosomal pathway to reduce progeny viral production. Our results reveal a previously undescribed role of Pin1 in the post-phosphorylation regulation of HBc and suggest that the Pin1 inhibition represents a promising new therapeutic option for treating HBV-related diseases.

The post-phosphorylation switch mediated by Pin1 is involved in the stability and function of several viral proteins. For example, Pin1 modulates DNA polymerase conformation of Epstein–Barr virus and is responsible for productive viral replication (Narita et al., 2013). Pin1 also binds to the non-structural NS5A/NS5B proteins of Hepatitis C virus, stabilizing them (Lim et al., 2011). The viral replication processes of HIV-1 in genome integration (Manganaro et al., 2010) and capsid uncoating (Misumi et al., 2010) are also regulated by Pin1. Moreover, Pin1 has been shown to enhance the stability of human T-cell leukemia virus type 1 Tax oncoprotein and facilitate the malignant transformation (Jeong et al., 2009). In the case of HBV infection, Pin1 binds HBx protein and increase its transcriptional competency to cell proliferation and oncogenesis (Pang et al., 2007). These studies demonstrate that Pin1 plays a pivotal function in viral replication for a broad range of viruses. However, the role of Pin1 in HBV replication, especially in viral core formation, has yet to be resolved. In our current report, we demonstrated that Pin1 also associate with phosphorylated HBc and stabilizes HBc, thereby promoting efficient virus propagation. Although we found that Pin1 suppressed HBc degradation through the inhibition of endo-lysosomal-mediated degradation pathway, its precise mechanism is still uncertain. A previous report showed that intracellular HBc proteins could be transported to early endosomes and lysosomes, depending on the adaptor protein Eps15 and the small GTPase Rab5 (Cooper and Shaul, 2006). Pin1 may prevent the association of these factors to HBc. Further careful analysis will be required to more precisely determine the molecular function of Pin1 with regard to HBc turnover during HBV particle production.

Hepatitis B virus core protein contains several phosphorylation recognition motifs at Ser or Thr residues preceding Pro (Ser/Thr-Pro) in its CTD phospho-acceptor sites, which are remarkably well conserved among related viruses (Jung et al., 2014). A previous report have identified at least seven conserved serine and threonine residues subjected to phosphorylated *in vivo* (Chen et al., 2011). Especially, the Ser-Pro motifs at positions 155, 162, and 170, are highly retained, and phosphorylated by multiple host serine/threonine protein kinases (Daub et al., 2002; Ludgate et al., 2012). The HBc protein contains another three major phosphorylated serine residues (Ser155, 162, and 170), along with four additional phosphorylated serine residues (Ser168, 176, and 178) and one phosphorylated threonine residue (Thr160) (Lan et al., 1999; Steven et al., 2005; Jung et al., 2014; Ludgate et al., 2016). By screening the Ser/Thr phosphorylation of HBc CTD using Phos-tag gel, we also identified two concomitant phosphorylations at Thr160 and Ser162, consistent with previous results (Jung et al., 2014; Heger-Stevic et al., 2018). CTD phosphorylation of HBc is mediated by host cell kinases, including cyclin-dependent kinase 2 (CDK2) (Ludgate et al., 2012), protein kinase C (PKC) (Kann and Gerlich, 1994), cyclin-dependent protein kinase p34^cdc2^ (also known as CDK1) (Yeh et al., 1993), the 46-kDa serine protein kinase (Kau and Ting, 1998), and serine/arginine-rich protein kinases 1 and 2 (SRPK1/2) (Daub et al., 2002; Heger-Stevic et al., 2018). PLK1 is also involved in CTD phosphorylation (Diab et al., 2017). However, it remains unclear whether phosphorylated HBc is conversely dephosphorylated by host phosphatases. Therefore, we screened a phosphatase library to uncover the molecular mechanism involved in the phosphorylation/dephosphorylation regulation of HBc. By screening 150 genes in the phosphatase library, we found that PDP2 interacts with phosphorylated HBc and dephosphorylates it, leading to HBc degradation and reduction of viral production. PDP2 dephosphorylates and reactivates the alpha subunit of the E1 component of the pyruvate dehydrogenase complex, and is thus involved in the enzymatic resetting of the pyruvate dehydrogenase complex (Huang et al., 1998). Therefore, it would be interesting to examine the relationship between glucose metabolism, HBc phosphorylation, and virus replication.

## Materials and Methods

### Cell Culture

HEK293 cells (ATCC, CRL-1573) and HepG2 cells (ATCC, HB-8065) were cultured in DMEM (Fujifilm Wako) containing 10% FBS. HepG2.2.15.7 cells (Iwamoto et al., 2017) were cultured with DMEM/F-12, GlutaMAX (Thermo Fisher Scientific) supplemented with 10% FBS, 10 mM HEPES (Thermo Fisher Scientific), and 5 μg/ml insulin (Sigma-Aldrich). HepG2 and HepG2.2.15.7 cells were grown on collagen-coated dishes.

### shRNA-Mediated Gene Silencing

To generate Pin1-depleted cells, cells were infected with retrovirus vector carrying Pin1-specific shRNA (Ryo et al., 2005). For the production of retroviruses, Plat-E cells (Morita et al., 2000) were transduced with pSUPER.retro vector and pVSV-G with Effectene reagent (Qiagen). After 48 h, cell supernatants were filtrated with a 0.45-μm filter and added with 10 μg/ml Polybrene. Target cells were then selected with 1 μg/ml puromycin (InvivoGen).

### GST Pull-Down, Immunoprecipitation, and Immunoblotting Analyses

GST pull-down assay was previously described (Nishi et al., 2011). Briefly, cells were treated with 100 nM bafilomycin A1 and 4 mM NH_4_Cl for 15 h before harvesting, treated with GST pull-down buffer (50 mM HEPES pH 7.4, 200 mM NaCl, 10% glycerol, 1% Triton X-100, 1.5 mM MgCl_2_, 1 mM EGTA, 1 mM EDTA, 100 mM NaF, 1 mM Na_3_VO_4_, 0.5 μg/ml leupeptin, 1 μg/ml pepstatin, and 0.2 mM PMSF), and incubated at 4°C for 3 h with glutathione-agarose beads containing either GST or GST-Pin1. The collected beads were then washed three times with GST pull-down buffer and processed for SDS-PAGE. To immunoprecipitate proteins, cells were harvested and lysed with NP-40 lysis buffer (10 mM Tris–HCl pH 7.4, 100 mM NaCl, 1% NP-40, 2 mM EDTA, 1% sodium deoxycholate, 50 mM NaF, 1 mM Na_3_VO_4_, 0.5 μg/ml leupeptin, 1 μg/ml pepstatin, and 0.2 mM PMSF). Cell lysates were then incubated for 1 h with protein A/G–Sepharose beads (GE Healthcare). Supernatant fractions were recovered and immunoprecipitated with 4 μg of mouse IgG or anti-HA (MBL) together with 20 μl of protein A/G–Sepharose at 4°C for 3 h. After washing three times with lysis buffer, the bound proteins were analyzed by immunoblotting, as previously described (Miyakawa et al., 2018, 2019). For Phos-tag PAGE, we used 12.5% acrylamide gel containing 50 μM Phos-tag (Fujifilm Wako). Source data are provided as a Supplementary Material (Data Sheets S1, Supplementary Material).

### Plasmids and Antibodies

The hepatitis B virus molecular clone pUC19-C_JPNAT (genotype C) has been described previously (Sugiyama et al., 2006). HBc cDNAs were amplified from pUC19-C_JPNAT with the appropriate primer pairs, followed by subcloning into the pcDNA-based N-HA vector (Thermo Fisher Scientific). The HBc derivatives were constructed using PCR-based mutagenesis. The primary antibodies used in this study were as follows: anti-HA (MBL), anti-FLAG and anti-α-tubulin (Sigma-Aldrich), anti-Pin1 (R&D System), anti-HaloTag (Promega), and anti-HBc monoclonal antibody (Kanto Chemical). A phospho-specific polyclonal antibody against HBc phosphorylated at Thr160 and Ser162 was generated by Scrum Inc. (Tokyo, Japan).

### Protein Degradation Assay

Protein degradation assays were performed as described previously (Nishi et al., 2011). Briefly, 100 μM cycloheximide was added to the medium, and the cells were harvested at the indicated time points. Total cell lysates in SDS sample buffer were boiled and analyzed by immunoblotting.

### Microscopic Analysis

Microscopic procedure was previously described (Miyakawa et al., 2017). Briefly, HepG2 cells were seeded onto glass cover slips 1 day before transfection. At 48 h post-transfection, the cells were fixed with 4% paraformaldehyde and permeabilized with 0.5% Triton X-100. The cells were then stained with anti-HA (MBL) and Alexa Fluor 488-conjugated secondary antibody (Thermo Fisher Scientific). For lysosome staining, cells were treated with Lysosomes-RFP reagents (Thermo Fisher Scientific) at 16 h prior to fixation. Microscopic imaging was performed with an FV1000-D confocal microscope (Olympus). Line plots of the fluorescence intensity were generated using the ImageJ software (NIH).

### NanoBRET-Based Protein–Protein Interaction Assays

Expression vectors encoding N-terminally HaloTag-conjugated host proteins (human phosphatases) were prepared by Kazusa Genome Technologies (Chiba, Japan) or purchased from Promega. NanoBRET analysis were performed as described previously (Miyakawa et al., 2019). Briefly, HEK293 cells were transfected with vectors encoding HaloTag-fused protein and NanoLuc-fused HBc at a 100:1 ratio. At 48 h post-transfection, NanoBRET activity was measured using the NanoBRET Nano-Glo Detection System (Promega).

### HBV Quantification Assays

Hepatitis B virus quantification procedure were previously described (Miyakawa et al., 2015). Culture supernatants of HepG2.2.15.7 cells or HepG2 cells expressing HBV molecular clone were cleared of cell debris by centrifugation at 3,000 rpm for 3 min. The HBcAg and HBeAg amounts in the culture supernatants were measured using HBcAg and HBeAg ELISA kit (Cell Biolabs), respectively. To remove the plasmid-derived DNA, culture supernatants were digested at 37°C for 2 h with 200 μg/ml DNase I, 100 μg/ml RNase A, and 6 mM MgOAc, and then centrifuged at 13,000 rpm for 1 min. The supernatants were then mixed with a buffer containing 10 mM EDTA, 1% SDS, 100 mM NaCl, and 200 μg/ml proteinase K (Roche), and incubated at 55°C for 1 h. These samples were extracted with phenol/chloroform, precipitated with ethanol, and dissolved in TE buffer (10 mM Tris–HCl pH 8.0, 1 mM EDTA). Amount of viral DNA was measured by real-time PCR using SYBR Premix Ex Taq II (Takara) as previously described (Miyakawa et al., 2015). For quantification of intracellular viral RNA, total RNA extraction was performed using the Trizol reagent (Thermo Fisher Scientific) and cDNA synthesis was conducted with ReverTra Ace (Toyobo), respectively.

## Data Availability Statement

The datasets generated for this study are available on request to the corresponding author.

## Author Contributions

MN and KM designed and performed the research, analyzed the data, and wrote the manuscript. SM and YY performed the research and analyzed the data. HKh analyzed the data and wrote the manuscript. KW and MS contributed reagents and analyzed the data. HKi and TW analyzed the data. AR directed the research, analyzed the data, and wrote the manuscript.

## Conflict of Interest

YY is a current employee of Kanto Chemical Co., Inc. The remaining authors declare that the research was conducted in the absence of any commercial or financial relationships that could be construed as a potential conflict of interest. The handling Editor declared a past co-authorship with authors KW and MS.

## Supplementary Material

DATA SHEET S1 Full images of the immunoblots presented in Figures 1–4.

DATA SHEET S2 Full images of the immunoblots presented in Figure 5.

## References

[B1] Baumert T. F. Thimme R. Von Weizsacker F. (2007). Pathogenesis of hepatitis B virus infection. *World J. Gastroenterol.* 13 82–90. 17206757 10.3748/wjg.v13.i1.82 PMC4065880

[B2] Beck J. Nassal M. (2007). Hepatitis B virus replication. *World J. Gastroenterol.* 13 48–64. 17206754 10.3748/wjg.v13.i1.48 PMC4065876

[B3] Brito A. F. Pinney J. W. (2017). Protein-protein interactions in virus-host systems. *Front. Microbiol.* 8:1557. 10.3389/fmicb.2017.01557 28861068 PMC5562681

[B4] Chao S. H. Greenleaf A. L. Price D. H. (2001). Juglone, an inhibitor of the peptidyl-prolyl isomerase Pin1, also directly blocks transcription. *Nucleic Acids Res.* 29 767–773. 10.1093/nar/29.3.767 11160900 PMC30403

[B5] Chen C. Wang J. C. Zlotnick A. (2011). A kinase chaperones hepatitis B virus capsid assembly and captures capsid dynamics in vitro. *PLoS Pathog.* 7:e1002388. 10.1371/journal.ppat.1002388 22114561 PMC3219723

[B6] Cooper A. Shaul Y. (2006). Clathrin-mediated endocytosis and lysosomal cleavage of hepatitis B virus capsid-like core particles. *J. Biol. Chem.* 281 16563–16569. 10.1074/jbc.m601418200 16618702

[B7] Cryer A. M. Imperial J. C. (2019). Hepatitis B in pregnant women and their infants. *Clin. Liver Dis.* 23 451–462. 10.1016/j.cld.2019.04.007 31266619

[B8] Daub H. Blencke S. Habenberger P. Kurtenbach A. Dennenmoser J. Wissing J. (2002). Identification of SRPK1 and SRPK2 as the major cellular protein kinases phosphorylating hepatitis B virus core protein. *J. Virol.* 76 8124–8137. 10.1128/jvi.76.16.8124-8137.2002 12134018 PMC155132

[B9] Diab A. Foca A. Fusil F. Lahlali T. Jalaguier P. Amirache F. (2017). Polo-like-kinase 1 is a proviral host factor for hepatitis B virus replication. *Hepatology* 66 1750–1765. 10.1002/hep.29236 28445592 PMC5658273

[B10] Diab A. Foca A. Zoulim F. Durantel D. Andrisani O. (2018). The diverse functions of the hepatitis B core/capsid protein (HBc) in the viral life cycle: implications for the development of HBc-targeting antivirals. *Antiviral Res.* 149 211–220. 10.1016/j.antiviral.2017.11.015 29183719 PMC5757518

[B11] Heger-Stevic J. Zimmermann P. Lecoq L. Bottcher B. Nassal M. (2018). Hepatitis B virus core protein phosphorylation: identification of the SRPK1 target sites and impact of their occupancy on RNA binding and capsid structure. *PLoS Pathog.* 14:e1007488. 10.1371/journal.ppat.1007488 30566530 PMC6317823

[B12] Huang B. Gudi R. Wu P. Harris R. A. Hamilton J. Popov K. M. (1998). Isoenzymes of pyruvate dehydrogenase phosphatase. DNA-derived amino acid sequences, expression, and regulation. *J. Biol. Chem.* 273 17680–17688. 10.1074/jbc.273.28.17680 9651365

[B13] Hunter T. (1995). Protein kinases and phosphatases: the yin and yang of protein phosphorylation and signaling. *Cell* 80 225–236. 10.1016/0092-8674(95)90405-0 7834742

[B14] Iwamoto M. Cai D. Sugiyama M. Suzuki R. Aizaki H. Ryo A. (2017). Functional association of cellular microtubules with viral capsid assembly supports efficient hepatitis B virus replication. *Sci. Rep.* 7:10620. 10.1038/s41598-017-11015-4 28878350 PMC5587681

[B15] Jeong S. J. Ryo A. Yamamoto N. (2009). The prolyl isomerase Pin1 stabilizes the human T-cell leukemia virus type 1 (HTLV-1) Tax oncoprotein and promotes malignant transformation. *Biochem. Biophys. Res. Commun.* 381 294–299. 10.1016/j.bbrc.2009.02.024 19338781

[B16] Jung J. Hwang S. G. Chwae Y. J. Park S. Shin H. J. Kim K. (2014). Phosphoacceptors threonine 162 and serines 170 and 178 within the carboxyl-terminal RRRS/T motif of the hepatitis B virus core protein make multiple contributions to hepatitis B virus replication. *J. Virol.* 88 8754–8767. 10.1128/JVI.01343-14 24850741 PMC4136252

[B17] Kann M. Gerlich W. H. (1994). Effect of core protein phosphorylation by protein kinase C on encapsidation of RNA within core particles of hepatitis B virus. *J. Virol.* 68 7993–8000. 10.1128/jvi.68.12.7993-8000.1994 7966589 PMC237262

[B18] Kau J. H. Ting L. P. (1998). Phosphorylation of the core protein of hepatitis B virus by a 46-kilodalton serine kinase. *J. Virol.* 72 3796–3803. 10.1128/jvi.72.5.3796-3803.1998 9557662 PMC109602

[B19] Kinoshita E. Kinoshita-Kikuta E. Takiyama K. Koike T. (2006). Phosphate-binding tag, a new tool to visualize phosphorylated proteins. *Mol. Cell. Proteomics* 5 749–757. 10.1074/mcp.t500024-mcp200 16340016

[B20] Kojima Y. Ryo A. (2010). Pinning down viral proteins: a new prototype for virus-host cell interaction. *Front. Microbiol.* 1:107. 10.3389/fmicb.2010.00107 21738521 PMC3125566

[B21] Lan Y. T. Li J. Liao W. Ou J. (1999). Roles of the three major phosphorylation sites of hepatitis B virus core protein in viral replication. *Virology* 259 342–348. 10.1006/viro.1999.9798 10388659

[B22] Lee H. W. Ahn S. H. (2016). Prediction models of hepatocellular carcinoma development in chronic hepatitis B patients. *World J. Gastroenterol.* 22 8314–8321. 27729738 10.3748/wjg.v22.i37.8314 PMC5055862

[B23] Lim Y. S. Tran H. T. Park S. J. Yim S. A. Hwang S. B. (2011). Peptidyl-prolyl isomerase Pin1 is a cellular factor required for hepatitis C virus propagation. *J. Virol.* 85 8777–8788. 10.1128/JVI.02533-10 21680504 PMC3165832

[B24] Liou Y. C. Zhou X. Z. Lu K. P. (2011). Prolyl isomerase Pin1 as a molecular switch to determine the fate of phosphoproteins. *Trends Biochem. Sci.* 36 501–514. 10.1016/j.tibs.2011.07.001 21852138 PMC3185210

[B25] Lu K. P. Finn G. Lee T. H. Nicholson L. K. (2007). Prolyl cis-trans isomerization as a molecular timer. *Nat. Chem. Biol.* 3 619–629. 10.1038/nchembio.2007.35 17876319

[B26] Lu K. P. Liou Y. C. Zhou X. Z. (2002). Pinning down proline-directed phosphorylation signaling. *Trends Cell Biol.* 12 164–172. 10.1016/s0962-8924(02)02253-5 11978535

[B27] Lu K. P. Zhou X. Z. (2007). The prolyl isomerase PIN1: a pivotal new twist in phosphorylation signalling and disease. *Nat. Rev. Mol. Cell Biol.* 8 904–916. 10.1038/nrm2261 17878917

[B28] Ludgate L. Liu K. Luckenbaugh L. Streck N. Eng S. Voitenleitner C. (2016). Cell-free hepatitis B virus capsid assembly dependent on the core protein C-terminal domain and regulated by phosphorylation. *J. Virol.* 90 5830–5844. 10.1128/JVI.00394-16 27076641 PMC4886785

[B29] Ludgate L. Ning X. Nguyen D. H. Adams C. Mentzer L. Hu J. (2012). Cyclin-dependent kinase 2 phosphorylates s/t-p sites in the hepadnavirus core protein C-terminal domain and is incorporated into viral capsids. *J. Virol.* 86 12237–12250. 10.1128/JVI.01218-12 22951823 PMC3486511

[B30] Machleidt T. Woodroofe C. C. Schwinn M. K. Mendez J. Robers M. B. Zimmerman K. (2015). NanoBRET–A novel BRET platform for the analysis of protein-protein interactions. *ACS Chem. Biol.* 10 1797–1804. 10.1021/acschembio.5b00143 26006698

[B31] Manganaro L. Lusic M. Gutierrez M. I. Cereseto A. Del Sal G. Giacca M. (2010). Concerted action of cellular JNK and Pin1 restricts HIV-1 genome integration to activated CD4+ T lymphocytes. *Nat. Med.* 16 329–333. 10.1038/nm.2102 20173753

[B32] McMahon B. J. (2005). Epidemiology and natural history of hepatitis B. *Semin. Liver Dis.* 25(Suppl. 1), 3–8. 16103976 10.1055/s-2005-915644

[B33] Misumi S. Inoue M. Dochi T. Kishimoto N. Hasegawa N. Takamune N. (2010). Uncoating of human immunodeficiency virus type 1 requires prolyl isomerase Pin1. *J. Biol. Chem.* 285 25185–25195. 10.1074/jbc.M110.114256 20529865 PMC2919081

[B34] Mitra B. Thapa R. J. Guo H. Block T. M. (2018). Host functions used by hepatitis B virus to complete its life cycle: implications for developing host-targeting agents to treat chronic hepatitis B. *Antiviral Res.* 158 185–198. 10.1016/j.antiviral.2018.08.014 30145242 PMC6193490

[B35] Miyakawa K. Matsunaga S. Watashi K. Sugiyama M. Kimura H. Yamamoto N. (2015). Molecular dissection of HBV evasion from restriction factor tetherin: a new perspective for antiviral cell therapy. *Oncotarget* 6 21840–21852. 26334101 10.18632/oncotarget.4808 PMC4673130

[B36] Miyakawa K. Matsunaga S. Yamaoka Y. Dairaku M. Fukano K. Kimura H. (2018). Development of a cell-based assay to identify hepatitis B virus entry inhibitors targeting the sodium taurocholate cotransporting polypeptide. *Oncotarget* 9 23681–23694. 10.18632/oncotarget.25348 29805766 PMC5955094

[B37] Miyakawa K. Matsunaga S. Yokoyama M. Nomaguchi M. Kimura Y. Nishi M. (2019). PIM kinases facilitate lentiviral evasion from SAMHD1 restriction via Vpx phosphorylation. *Nat. Commun.* 10:1844. 10.1038/s41467-019-09867-7 31015445 PMC6479052

[B38] Miyakawa K. Nishi M. Matsunaga S. Okayama A. Anraku M. Kudoh A. (2017). The tumour suppressor APC promotes HIV-1 assembly via interaction with Gag precursor protein. *Nat. Commun.* 8:14259. 10.1038/ncomms14259 28134256 PMC5290283

[B39] Morita S. Kojima T. Kitamura T. (2000). Plat-E: an efficient and stable system for transient packaging of retroviruses. *Gene Ther.* 7 1063–1066. 10.1038/sj.gt.3301206 10871756

[B40] Nakamura K. Greenwood A. Binder L. Bigio E. H. Denial S. Nicholson L. (2012). Proline isomer-specific antibodies reveal the early pathogenic Tau conformation in Alzheimer’s disease. *Cell* 149 232–244. 10.1016/j.cell.2012.02.016 22464332 PMC3601591

[B41] Narita Y. Murata T. Ryo A. Kawashima D. Sugimoto A. Kanda T. (2013). Pin1 interacts with the Epstein-Barr virus DNA polymerase catalytic subunit and regulates viral DNA replication. *J. Virol.* 87 2120–2127. 10.1128/JVI.02634-12 23221557 PMC3571458

[B42] Nassal M. (1992). The arginine-rich domain of the hepatitis B virus core protein is required for pregenome encapsidation and productive viral positive-strand DNA synthesis but not for virus assembly. *J. Virol.* 66 4107–4116. 10.1128/jvi.66.7.4107-4116.1992 1602535 PMC241213

[B43] Newman M. Suk F. M. Cajimat M. Chua P. K. Shih C. (2003). Stability and morphology comparisons of self-assembled virus-like particles from wild-type and mutant human hepatitis B virus capsid proteins. *J. Virol.* 77 12950–12960. 10.1128/jvi.77.24.12950-12960.2003 14645551 PMC296082

[B44] Nishi M. Akutsu H. Masui S. Kondo A. Nagashima Y. Kimura H. (2011). A distinct role for Pin1 in the induction and maintenance of pluripotency. *J. Biol. Chem.* 286 11593–11603. 10.1074/jbc.M110.187989 21296877 PMC3064213

[B45] Pang R. Lee T. K. Poon R. T. Fan S. T. Wong K. B. Kwong Y. L. (2007). Pin1 interacts with a specific serine-proline motif of hepatitis B virus X-protein to enhance hepatocarcinogenesis. *Gastroenterology* 132 1088–1103. 10.1053/j.gastro.2006.12.030 17383430

[B46] Ryo A. Liou Y. C. Lu K. P. Wulf G. (2003). Prolyl isomerase Pin1: a catalyst for oncogenesis and a potential therapeutic target in cancer. *J. Cell Sci.* 116 773–783. 10.1242/jcs.00276 12571275

[B47] Ryo A. Uemura H. Ishiguro H. Saitoh T. Yamaguchi A. Perrem K. (2005). Stable suppression of tumorigenicity by Pin1-targeted RNA interference in prostate cancer. *Clin. Cancer Res.* 11 7523–7531. 10.1158/1078-0432.ccr-05-0457 16243827

[B48] Steven A. C. Conway J. F. Cheng N. Watts N. R. Belnap D. M. Harris A. (2005). Structure, assembly, and antigenicity of hepatitis B virus capsid proteins. *Adv. Virus Res.* 64 125–164. 10.1016/s0065-3527(05)64005-5 16139594

[B49] Sugiyama M. Tanaka Y. Kato T. Orito E. Ito K. Acharya S. K. (2006). Influence of hepatitis B virus genotypes on the intra- and extracellular expression of viral DNA and antigens. *Hepatology* 44 915–924. 10.1002/hep.21345 17006908

[B50] Wang M. Xi D. Ning Q. (2017). Virus-induced hepatocellular carcinoma with special emphasis on HBV. *Hepatol. Int.* 11 171–180. 10.1007/s12072-016-9779-5 28097530

[B51] Wulf G. Finn G. Suizu F. Lu K. P. (2005). Phosphorylation-specific prolyl isomerization: is there an underlying theme? *Nat. Cell Biol.* 7 435–441. 10.1038/ncb0505-435 15867923

[B52] Yeh C. T. Wong S. W. Fung Y. K. Ou J. H. (1993). Cell cycle regulation of nuclear localization of hepatitis B virus core protein. *Proc. Natl. Acad. Sci. U.S.A.* 90 6459–6463. 10.1073/pnas.90.14.6459 8341655 PMC46951

[B53] Zheng C. L. Fu Y. M. Xu Z. X. Zou Y. Deng K. (2019). Hepatitis B virus core protein dimerdimer interface is critical for viral replication. *Mol. Med. Rep.* 19 262–270. 10.3892/mmr.2018.9620 30387827 PMC6297743

[B54] Zhou X. Z. Kops O. Werner A. Lu P. J. Shen M. Stoller G. (2000). Pin1-dependent prolyl isomerization regulates dephosphorylation of Cdc25C and Tau proteins. *Mol. Cell* 6 873–883. 10.1016/s1097-2765(05)00083-3 11090625

