# Supplementary material for: Prolyl Isomerase Pin1 Regulates the Stability of Hepatitis B Virus Core Protein
Source: Front Cell Dev Biol. 2020 Jan 31;8:26. doi: 10.3389/fcell.2020.00026 (PMC7005485; doi:10.3389/fcell.2020.00026)

Fig.1B

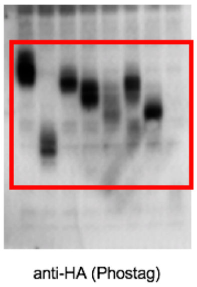

Fig.1C

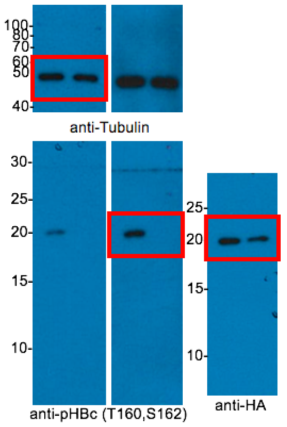

Fig.1D

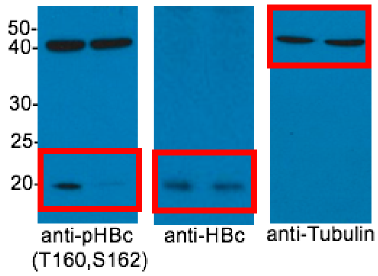

Fig.2A

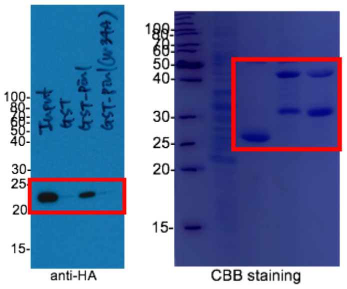

Fig.2B

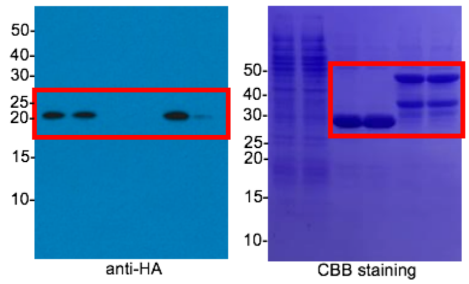

Fig.2C

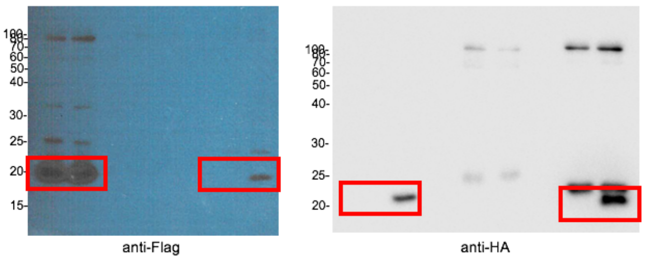

Fig.2D

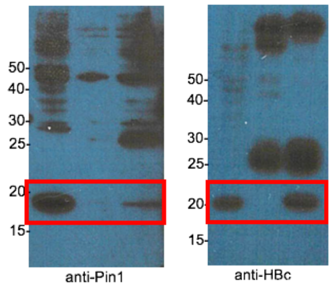

Fig.2E

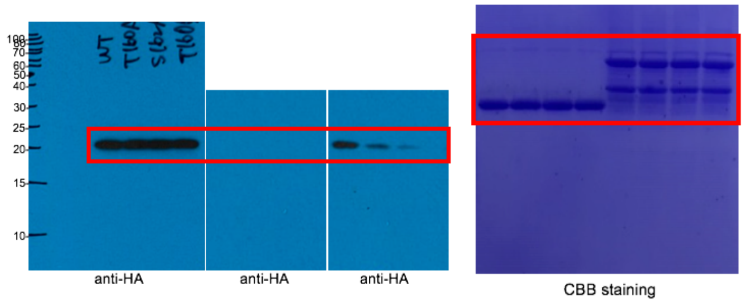

Fig.2F

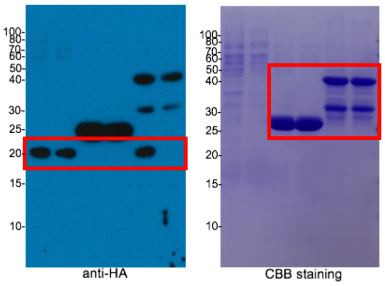

Fig.2G

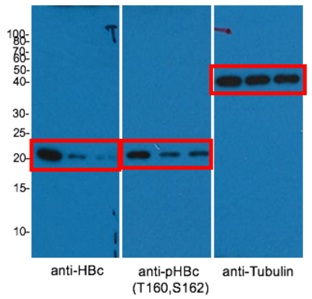

Fig.3A

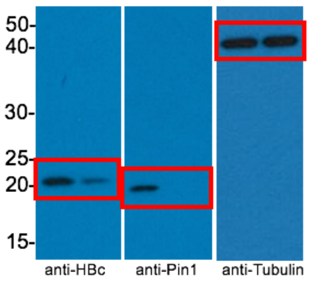

Fig.3C

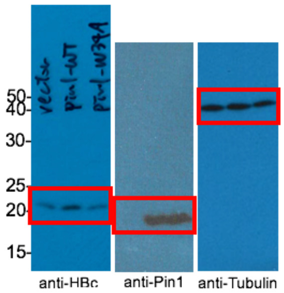

Fig.3D

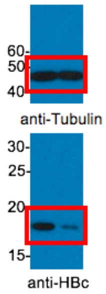

Fig.3E

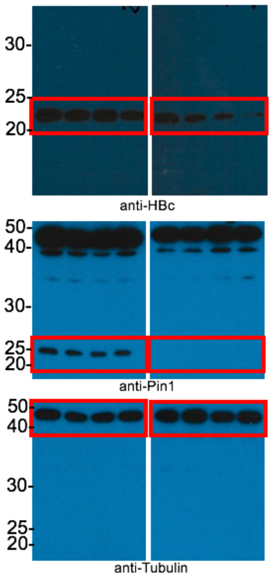

Fig.3F

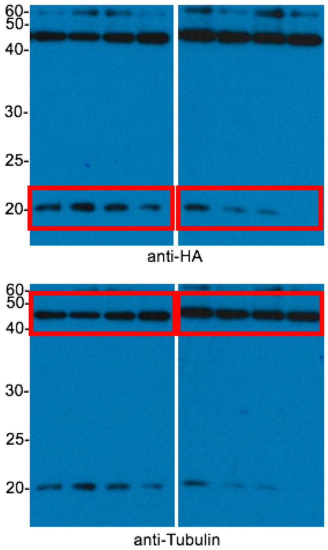

Fig.4A

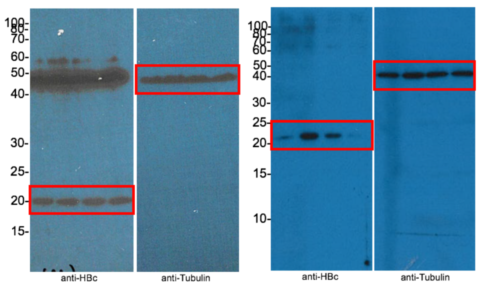

Supplement: DATA SHEET S1 — Full images of the immunoblots presented in Figures 1–4. [file Data_Sheet_1.PDF]
