# Supplementary figures and images for: Prolyl Isomerase Pin1 Regulates the Stability of Hepatitis B Virus Core Protein
Source: Front Cell Dev Biol. 2020 Jan 31;8:26. doi: 10.3389/fcell.2020.00026 (PMC7005485; doi:10.3389/fcell.2020.00026)

Fig.5B

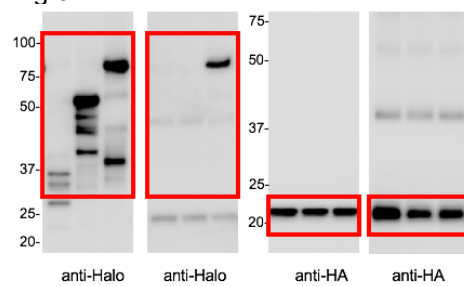

Fig.5C

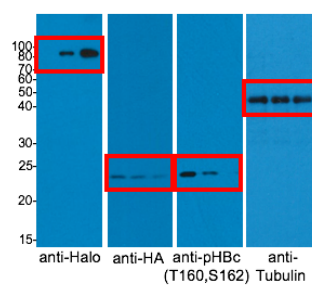

Fig.5D

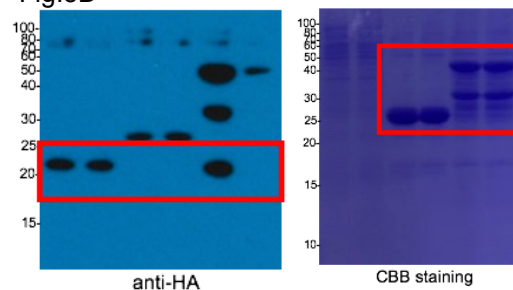

Fig.5E

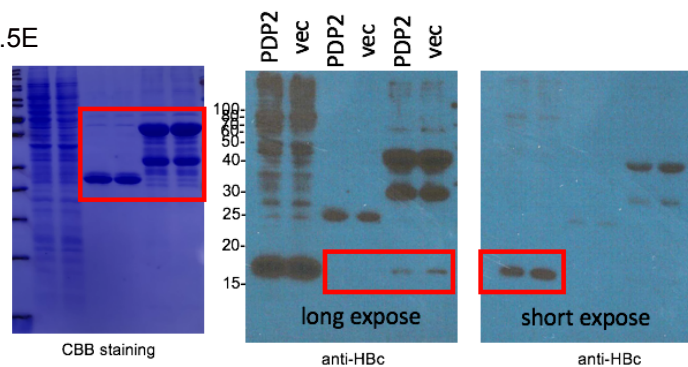

Fig.5F

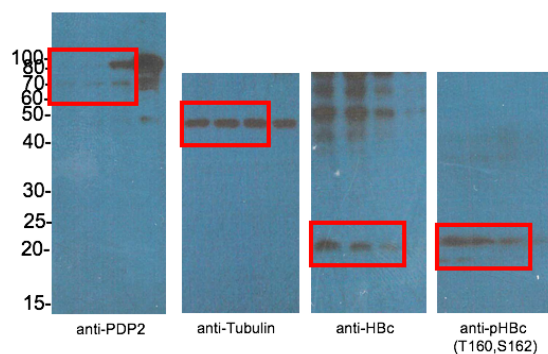

Supplement: DATA SHEET S2 — Full images of the immunoblots presented in Figure 5. [file Data_Sheet_2.PDF]
